# Cholesterol Tuning of BK Ethanol Response Is Enantioselective, and Is a Function of Accompanying Lipids

**DOI:** 10.1371/journal.pone.0027572

**Published:** 2011-11-29

**Authors:** Chunbo Yuan, Maohui Chen, Douglas F. Covey, Linda J. Johnston, Steven N. Treistman

**Affiliations:** 1 Institute of Neurobiology, University of Puerto Rico, San Juan, Puerto Rico; 2 Steacie Institute for Molecular Sciences, National Research Council of Canada, Ottawa, Ontario, Canada; 3 Department of Developmental Biology, Washington University School of Medicine, St. Louis, Missouri, United States of America; University of Michigan, United States of America

## Abstract

In the search to uncover ethanol's molecular mechanisms, the calcium and voltage activated, large conductance potassium channel (BK) has emerged as an important molecule. We examine how cholesterol content in bilayers of 1,2-dioleoyl-3-phosphatidylethanolamine (DOPE)/sphingomyelin (SPM) and 1-palmitoyl-2-oleoyl-sn-glycero-3-phosphatidylethanolamine (POPE)/1-palmitoyl-2-oleoyl-sn-glycero-3-phosphatidylserine (POPS) affect the function and ethanol sensitivity of BK. In addition, we examine how manipulation of cholesterol in biological membranes modulates ethanol's actions on BK. We report that cholesterol levels regulate the change in BK channel open probability elicited by 50 mM ethanol. Low levels of cholesterol (<20%, molar ratio) supports ethanol activation, while high levels of cholesterol leads to ethanol inhibition of BK. To determine if cholesterol affects BK and its sensitivity to ethanol through a direct cholesterol-protein interaction or via an indirect action on the lipid bilayer, we used the synthetic enantiomer of cholesterol (*ent-*CHS). We found that 20% and 40% *ent-*CHS had little effect on the ethanol sensitivity of BK, when compared with the same concentration of *nat-*CHS. We accessed the effects of *ent*-CHS and *nat*-CHS on the molecular organization of DOPE/SPM monolayers at the air/water interface. The isotherm data showed that *ent*-CHS condensed DOPE/SPM monolayer equivalently to *nat*-CHS at a 20% concentration, but slightly less at a 40% concentration. Atomic force microscopy (AFM) images of DOPE/SPM membranes in the presence of *ent*-CHS or *nat*-CHS prepared with LB technique or vesicle deposition showed no significant difference in topographies, supporting the interpretation that the differences in actions of *nat-*CHS and *ent*-CHS on BK channel are not likely from a generalized action on bilayers. We conclude that membrane cholesterol influences ethanol's modulation of BK in a complex manner, including an interaction with the channel protein. Finally, our results suggest that an understanding of membrane protein function and modulation is impossible unless protein and surrounding lipid are considered as a functional unit.

## Introduction

For many years, lipid perturbation was considered to be the primary molecular mechanism responsible for the actions of ethanol in the nervous system, responsible for downstream effects on protein function [1]. However, data collected more recently, especially using mutagenesis, have shifted the focus of the ethanol field to the direct actions of ethanol on membrane proteins [2], [3], [4]. However, work using highly reduced systems in which ethanol's actions are analyzed in bilayers containing only a small number of lipids and a single species of membrane protein channel have made very clear that even after accepting the proposition that target proteins contain a binding site for ethanol, the lipids adjacent to the protein exert a profound influence on the response of the protein to the drug. Thus, any interpretation of drug action at the molecular level that considers the protein, but does not consider the contribution of its lipid environment is likely to be inadequate [5]. Among the attributes of lipids that influence ethanol's actions on imbedded proteins are bilayer thickness, charge, and headgroup identity [6], [7], [8]. Cholesterol is a membrane sterol that has been shown to exert a particularly powerful influence on the response of membrane proteins to ethanol.

Cholesterol is a major component, in addition to phospholipids, sphingolipids and glycolipids [9] of cell membranes. It is essential not only in maintaining cell membrane structures [10], [11] such as lipid (or membrane) rafts [12], but also in the physiology and function of membrane ion channels and receptors [13], [14], [15], [16]. As would be expected, cholesterol levels in membranes are maintained within a narrow range by sophisticated and complex mechanisms [17]. When these homeostatic mechanisms are overwhelmed, the consequences can be severe, causing a variety of pathological conditions [12].

Changes in membrane cholesterol levels alter the function of a number of ion channels. For example, cholesterol depletion enhances activity of inwardly rectifying K^+^ (Kir) channels [18], [19], Ca^2+^-sensitive K^+^ channels [20], [21], [22], N-type Ca^2+^ channels [23], and volume-regulated anion channels [15]. In contrast, epithelial Na^+^ channels and Ca^2+^-sensitive K^+^ channels in glioma cells are inhibited by cholesterol depletion [24], [25]. The mechanism underlying cholesterol modulation of ion channels is not understood. Generally, cholesterol can affect an ion channel through either: 1) direct interaction with the target channel or its associated proteins, or 2) indirect interaction via perturbation of the lipid bilayer surrounding the protein. Enantiomeric cholesterol (*ent-*CHS) [26] has been used [27], [28] to differentiate direct protein interaction from indirect mediation via lipids. *ent-*CHS is a chemically synthesized cholesterol [29] that has chemical composition identical to natural cholesterol (*nat-*CHS) but differs in absolute configuration for each stereocenter. It has been shown to have identical effects on the biophysical properties of several different lipid mono- and bilayers, but its interaction with some membrane proteins differs from that of *nat-*CHS [30]. Indeed, a strategy using *ent-*CHS has very recently been applied to understanding the actions of cholesterol on BK channel activity, with the authors concluding that cholesterol interacts specifically with the channel protein [31].

In addition to the role played by membrane cholesterol in the function of the large conductance, Ca^2+^-sensitive K^+^ channel (BK) [31], we have reported that it has profound effects on the ethanol response of BK. Inclusion of 30% cholesterol in POPE/POPS bilayers dramatically reduces BK channel activity, and effectively antagonizes ethanol potentiation of the channel [6]. In addition, physical properties of lipid membranes (such as bilayer thickness) affect BK channel activity [32] and the development of acute tolerance to ethanol [8].

Interestingly, BK channel protein incorporated into DOPE/SPM bilayers does not exhibit the ethanol activation seen in all other lipid bilayers tested (POPE/POPS and PC from PC 14 to PC 24) [8]. This lipid mixture is particularly interesting because cholesterol and sphingomyelin form lipid microdomains that are frequently used to model the behavior of lipid rafts in natural membranes [33], [34], [35], [36], [37], [38]. Indeed, the DOPE/SPM bilayer was unique among a series of bilayers of different thicknesses, with incorporated BK not only exhibiting no potentiation, but instead, showing an inhibitory response to ethanol [32]. We singled out this lipid mixture to investigate the influence of cholesterol on channel activity and ethanol sensitivity. Our results indicate that bilayer cholesterol can function as a toggle regulating the channel's response to ethanol in the DOPE/SPM bilayer. Incorporation of small amount of cholesterol (20% cholesterol, molar ratio) into DOPE/SPM bilayers reverses the action of ethanol from BK inhibition seen in the absence of cholesterol [8], to potentiation of the channel. When the cholesterol concentration is greater than 30% (molar ratio), the channel again becomes inhibited by ethanol. Further, experiments using enantiomeric cholesterol indicate that cholesterol's modulation of BK's ethanol response results from a specific interaction between cholesterol and the channel protein. This complex tuning of the ethanol response by lipids suggests a potential biological strategy in the development of tolerance to ethanol by lateral movement of critical molecules such as BK within the plane of the membrane, to domains in which the response to the drug is reduced.

## Materials and Methods

### Materials

POPE, POPS, DOPE, brain SPM and natural cholesterol were obtained from Avanti Polar Lipids (Alabaster, AL). They were used without further purification. Enantiomeric cholesterol was synthesized as described before [39]. Decane and salts were from Aldrich Chem. Co. Inc. (St. Louis, MO). Ethanol (100%, anhydrous) was purchased from American Bioanalytical (Natick, MA). All aqueous solutions were prepared with 18.3 MΩ/cm Milli-Q water (Millipore Corp., Billerica, MA).

### Membrane preparation

The cDNA encoding hSlo, kindly provided by Dr. P. Ahring, NeuroSearch A/S, (Copenhagen, Denmark) was over expressed in HEK-293 cells [40]. Stably transfected HEK-293 cells were grown in artificial medium and membrane fragments were prepared using a protocol developed for COS cells [41] with some slight modifications as described elsewhere [6].

### Monolayer preparation and isotherms

Monolayers of DOPE, SM and cholesterol mixtures were prepared by Langmuir-Blodgett (LB) deposition using a Langmuir-Blodgett trough (NIMA Model 611, Coventry, UK) equipped with a movable barrier and a Wilhelmy surface balance. All monolayers were prepared in a nitrogen-purged box, <1% oxygen, in order to avoid lipid oxidation. The mixtures were dissolved in chloroform/methanol to give a 1 mg/mL stock solution. A 20 µL volume of stock solution was spread at the interface. The solvent was allowed to evaporate (10 min), and the compression isotherm was recorded at a compression rate of 50 cm^2^/min. The monolayers were annealed by two compression/expansion cycles before transfer onto the ∼1 inch×1 inch freshly cleaved mica at the desired surface pressure (10 and 30 mN/m). The dipping speed was 5 mm/min upstroke. The monolayers were allowed to dry for at least 30 min, and then used for further experiments.

### Bilayers from Vesicle Fusion

DOPE/SPM (3/2 molar ratio) mixture with 20 mol % or 40 mol % cholesterol was dissolved in chloroform/methanol (4∶1 ratio, 1 mg/mL) in a small vial and then dried under a stream of nitrogen and held under high vacuum overnight to form dry lipid films. Small unilamellar vesicles (SUVs, 1 mg/mL) were prepared by swelling the lipid films with Milli-Q water and then sonicating in a bath sonicator (VWR, B1500A-DTH) at ∼45°C for 60 min. Supported bilayers were formed by adding 25 µL of vesicle solution and 475 µL of buffer (150 mM KCl, 1.03 mM CaCl2, 1.1 mM EDTA, 10 mM HEPES, pH 7.2) to freshly cleaved mica in a liquid cell. The sample was incubated at 45°C for 1 hr, followed by gradually cooling to room temperature with a controlled cooling rate. The bilayers were extensively rinsed with buffer solution to remove unattached vesicles before AFM imaging.

### AFM Measurements

Tapping mode AFM imaging for both monolayers and bilayers was carried out at room temperature using a PicoSPM atomic force microscope (Molecular Imaging). Magnetic coated silicon tips (MAC Levers type II, Agilent) with spring constants of ∼2.8 N/m were used. A 30×30 µm^2^ scanner was operated at a scan rate between 0.8 and 1.2 Hz. The images shown are flattened raw data.

### Electrophysiology

HEK 293 cells stably expressing hSlo α channels were cultured in 35-mm culture dishes until 45–60% confluence was reached. Cells were washed for 30 min in high calcium (2.2 mM) bath solution followed by 10-min wash in intracellular recording solution (1 µM calcium) prior to recording. Recordings of whole cell current followed the standard patch-clamp techniques. All recordings were made under symmetric K+ conditions where the potassium concentration was the same in the bath and recording solutions.

Electrodes were fabricated from glass pipettes (Drummond Scientific, Broomall, PA), pulled using a Model P-97 Brown/Flaming micropipette puller (Sutter Instrument, Novato, CA), and coated with sylgard (Dow Corning, Midland, MI) to reduce capacitance and noise. The tips were fire polished using a microforge (Narishige, Kyoto, Japan) to yield electrodes with resistances between 7 and 15 MΩ when filled with high K^+^ extracellular recording solution. An agar bridge containing an Ag/AgCl pellet and 3% agar in buffer solution was used as a ground. Single-channel currents were recorded using an EPC-9 (HEKA Elektronik, Lambrecht, Germany) patch-clamp amplifier at a bandwidth of 3 kHz and were low-pass filtered at 1 kHz using an eight pole Bessel filter (model 902LPF, Frequency Devices, Haverhill, MA) and sampled at 5 kHz using PatchMaster software. Data were acquired and stored using an A/D converter and a Dell Computer. Single-channel conductances were obtained from I/V plots. Each patch was recorded at a given voltage from −40 to +80 for 800 milliseconds. To obtain the time course of whole-cell current in response to the treatment of MβCD and the application of 50 mM ethanol, the whole-cell current was sampled for 13 seconds at one-minute intervals.

### Solutions

The high-calcium bath solution contained (in mM) 135 Na^+^ gluconate, 5 K^+^ gluconate, 2.2 CaCl_2_, 1 MgCl_2_, and 15 HEPES. The high-K+ extracellular recording solution contained (in mM) 140 K^+^ gluconate, 2.2 CaCl_2_, 4 EGTA, 4 HEDTA, 1 MgCl_2_, and 15 HEPES. The 1-µM calcium intracellular recording solution contained (in mM) 140 K^+^ gluconate, 5 Na^+^ gluconate, 0.43 CaCl_2_, 2 HEDTA, 1 MgCl_2_, and 15 HEPES. Solutions were brought to pH 7.35 with KOH or NaOH as needed. These conditions were optimized for seal formation and patch stability.

### Depletion of membrane cholesterol

For treatments using methyl-ß-cyclodextrin (MßCD), MßCD at a concentration of 5 mg/ml was dissolved in the 1-µM calcium intracellular recording solution, and was perfused onto cells for recording time of 15 min.

### Planar Bilayer recording

Single channel recordings were carried out with standard planar bilayer technology. Binary lipid mixtures of POPE/POPS (3∶1, molar ratio) and DOPE/SPM (3/2, molar ratio) were initially dissolved in chloroform. The solvent was removed by evaporation with a N_2_ stream and then the dried lipid film was resuspended in decane to form a final total lipid concentration of 25 mg/ml. The bilayer was formed by painting the lipid solution across a 250 µm aperture in a Delrin bilayer chamber (model CD-P250 from Warner Instruments, Hamden CT). Bilayer capacitance was monitored by noting the current across the bilayer in response to a triangle wave (10 mV/25 ms). Membrane suspensions containing crude membrane fragments (0.2–0.5 µl) were directed to the bilayer in the cis chamber with a micropipette. The cytoplasmic cis solution contained: 300 mM KCl, 1.03 mM CaCl_2_, 1.1 mM HEDTA, 10 mM HEPES, pH 7.2. The free Ca^2+^ was measured with a Ca^2+^ electrode to be about 20 µM. Ca^2+^ standard solutions were from World Precision Instruments (Sarasota, FL). Additional drops of 0.1 M HEDTA were added to the cis chamber to lower free Ca^2+^ concentration (in the range of 8∼15 µM) so that a low nP_o_ (in the range of 0.05∼0.2) could be achieved. This allowed us to study ethanol potentiation of BK_Ca_ channels in lipid bilayers of POPE/POPS and DOPE/SPM. The recording solutions were modified from those used in the patch experiments to obtain stable bilayer recordings and the free Ca^2+^ concentrations were chosen to get a modest channel open probability (P_o_). Ethanol was added as pure ethanol to the cis buffer in amounts necessary to reach the desired 50 mM concentration. Vigorous mixing of the buffer solutions was achieved by continuous stirring of both chambers with a stir bar at its full power (Sun Stir3 from Warner Instruments). The extracellular (trans) solution in the inner chamber contained: 150 mM KCl, 0.1 mM HEDTA, 10 mM HEPES, pH 7.2. Single channel currents were recorded with a patch-clamp amplifier (EPC-9, HEKA Elektronik, Lambrecht, Germany) [42]. The trans chamber was connected to ground and all voltages in the cis chamber were expressed relative to ground. The holding potential was usually at 20 mV unless stated otherwise. At least one minute of recording was taken as a control after insertion to ensure stable channel activity before application of ethanol. Continuous recordings were taken after the application of ethanol to obtain the time course of the ethanol response. All experiments were done at room temperature (22 °C).

*Data analysis:* nP_o_ was calculated as an index of steady-state channel activity from the all-points histogram and the number of channels (n) in the recording and the open channel probability (P_o_) as described elsewhere [43]. Data are expressed as mean ± S.E.M. All analyses were done with TAC and TAC-fit programs (Bruxton Corporation, Seattle, WA). Statistical analysis was conducted with Origin 7 (OriginLab, Northampton, MA). Data were analyzed by standard ANOVA and Tukey honest significant difference tests. Statistical significance for all of these tests was set at p<0.05.

## Results

### Cholesterol modulates BK's ethanol response in a concentration dependent manner in DOPE/SPM bilayers

**Figure 1:**
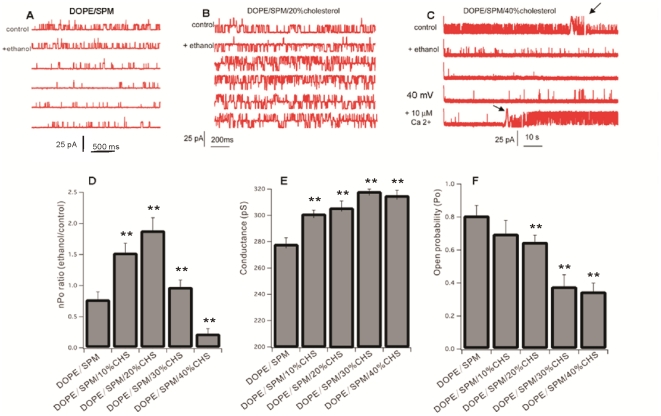
Sample single channel records of BK current recorded in (A) DOPE/SPM, (B) DOPE/SPM with 20% cholesterol, or (C) DOPE/SPM with 40% cholesterol. In each case, the response to 50 mM ethanol is shown. Ethanol consistently failed to activate BK in the DOPE/SPM bilayer, while in the presence of 20% cholesterol, 50 mM ethanol potentiated the channel activity in 5 of 5 experiments (average activation about 2-fold). Higher cholesterol levels, however, resulted in a reversal of ethanol action, with strong inhibition observed in 9 of 9 experiments (C), The arrows in the Fig. 1C show where 50 mM ethanol and 10 µM Ca2+ were added. Only one channel was recorded in the bilayer, and the spikes seen in the figure (marked as arrows) were due to turning of the stir bar. A fuller range of cholesterol values and its influence on ethanol action, conductance, and gating is shown graphically in D–F. **, significantly different from control (P<0.01).

Fig. 1 shows single channel recordings of 50 mM ethanol applied to a BK channel in a DOPE/SPM bilayer, and in DOPE/SPM bilayers containing 20% or 40% cholesterol. Ethanol failed to activate BK repeatedly (9 of 11 times) in DOPE/SPM bilayers [8] (see Fig. 1A). However, addition of 20% cholesterol to the DOPE/SPM membrane supported ethanol potentiation of BK (Fig. 1 B). Activation was observed in 5 out of 5 bilayer recordings. The average nPo ratio (ethanol/control nPo) of BK in the presence of 20% cholesterol was 1.89±0.23, which is similar to the values observed for BK channels in lipid bilayers of PC 18 to PC 22 [8]. When cholesterol content was increased to 40% of total lipid, single BK channel activity in DOPE/SPM/cholesterol bilayers was dramatically inhibited by ethanol. Fig. 1C shows a representative current recording of a single BK channel in a DOPE/SPM membrane containing 40% cholesterol. The presence of 40% cholesterol reduced the channel activities, mostly by reducing the channel open time, and by increasing the shut time, which is consistent with the previous reports [6], [44], in which cholesterol was inserted into POPE/POPS bilayers. Exposure to 50 mM ethanol decreased channel activity immediately and within one minute the channel was totally silent. To confirm that the silenced single channel was still in the membrane, we depolarized to +40 mV, whereupon activity returned. When the free Ca2+ concentration was raised to 25 µM, open probability (Po) reached 0.85, and indicated one channel in the bilayer. The inhibition of BK channel activity by ethanol was observed 9 out of 9 times in DOPE/SPM membranes containing 40% cholesterol. The average nPo ratio (ethanol/control nPo) was 0.24±0.08. Clearly, the ethanol sensitivity of BK in DOPE/SPM membranes is selectively regulated by different cholesterol concentrations. Extending the tested cholesterol concentrations in DOPE/SPM membranes to include 10% and 30% cholesterol (Fig. 1D), indicates that while 10% cholesterol supports potentiation, when the cholesterol concentration reaches 30%, BK begins to be inhibited. Thus, the sign (potentiation vs. inhibition) of BK channel response to ethanol is responsive to the level of membrane cholesterol. Other parameters of BK function, such as single channel conductance (Gc) and open probability (Po), were monotonically modulated by membrane cholesterol level. Consistent with results previously observed in POPE/POPS bilayers [6], increased cholesterol in the DOPE/SPM bilayer reduced the single channel open probability and increased slightly the channel conductance (see Fig. 1E and F).

### Depletion of membrane cholesterol in HEK 293 cells increases BK current, and alters BK response to ethanol

**Figure 2:**
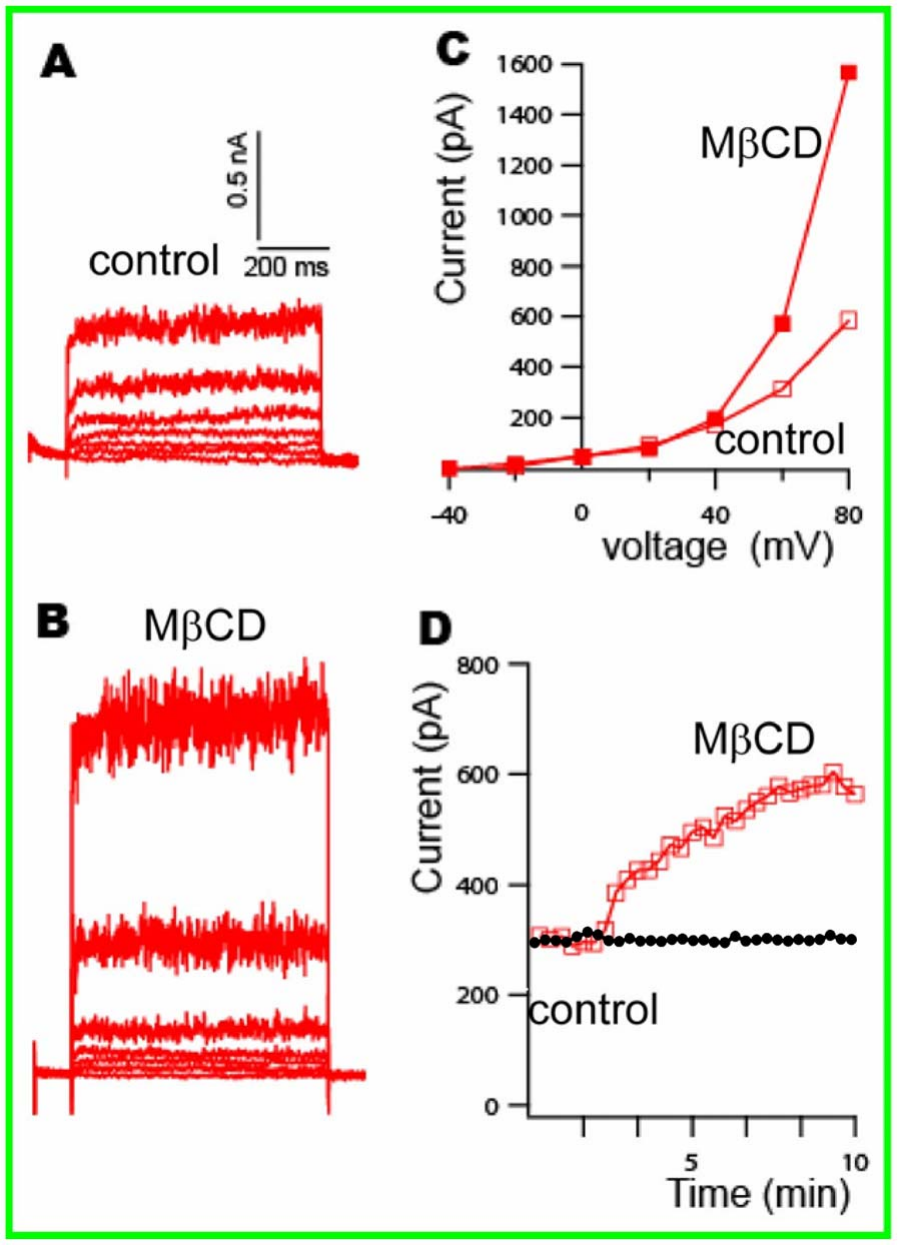
Whole-cell current recordings of BK in HEK 293 cells. Depletion of cholesterol by MßCD treatment increased BK current dramatically. (A) Control records of BK current in HEK 293 cells stepped from a holding potential of −70 mV to −40 mV∼+80 mV. (B) The same voltage protocol applied to the same cell after cholesterol depletion by MßCD. (C) shows compiled current-voltage plots before and after MßCD treatment. (D) Data showing control data of stable BK current without MßCD treatment and sequential values of BK current over time, before and after MßCD treatment at the holding potential of +60 mV.

We next examined how manipulation of cholesterol content in biological membranes affects BK channel activity and its ethanol sensitivity in HEK cells. The HEK 293 cells used in our experiments over-expressed hSlo alpha BK channels. We have confirmed the BK current in HEK cells by: 1) the large single channel conductance (>220 pS); 2) the currents were sensitive to free Ca2+; and 3) the currents were sensitive to membrane holding potentials [8]. Even though HEK293 cells do contain endogenous delayed and outwardly rectifying potassium currents, those currents are very small. We used 5 mM methyl-β-cyclodextrin (MβCD) to selectively deplete membrane cholesterol in the HEK cell membrane [25]. Fig. 2 shows whole-cell current recordings of BK in HEK 293 cells transfected with BKα (see Methods), before and after treatment with 5 mM MβCD. An increase in whole-cell current (2–5 fold) by depletion of membrane cholesterol is evident (Fig. 2C). This result differs from findings obtained for BK channels in glioma cells [25] but is consistent with previously published bilayer data [6], as well as data obtained in IGR 39 cells [45]. The time course of current rise is gradual, with maximal current observed at about 10 minutes after the application of MβCD (Fig. 2D).

**Figure 3:**
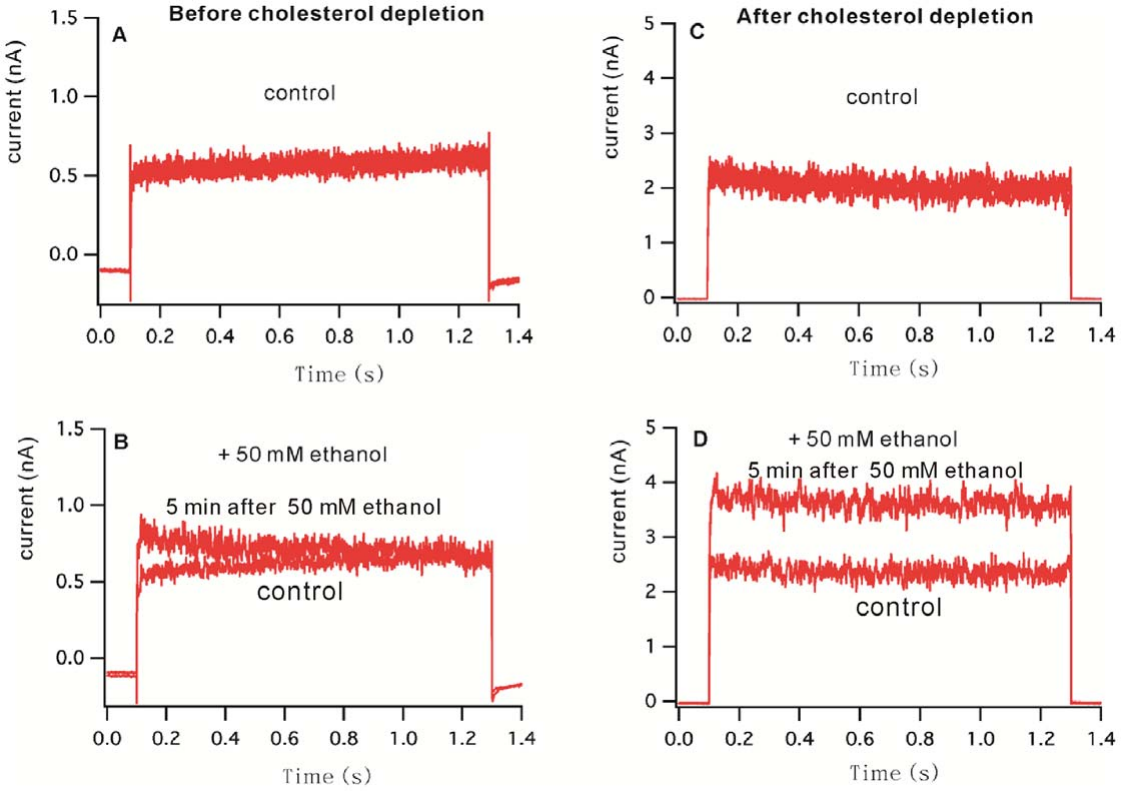
Whole-cell current of BK channel in HEK 293 cells before (A, B) and after (C, D) MßCD treatment, showing the effect of cholesterol depletion on the response of BK channel to exposure of 50 mM ethanol. The whole cell currents for the controls and those after ethanol applications were sampled for 13 second at 1 minute interval. Figure A and C are the control records showing 5 consecutive sampling of current, overlapped each other, displaying a stable current before exposure to 50 mM ethanol, B and D showed the currents before exposure to 50 mM ethanol and after exposure to ethanol at about 5 minutes. The current was recorded at a membrane voltage of +60 mV. After depletion of membrane cholesterol, the response to ethanol is more robust and long lasting, compared to before depletion, in which ethanol activation of BK is transient and less robust.

We tested ethanol's effect on BK in HEK 293 cells before and after treatment with MβCD. A sample recording of whole cell currents is shown in Fig. 3. The average whole-cell current before MβCD treatment is about 0.5 nA at +60 mV, and is stable over time (see Fig. 3A). Application of 50 mM ethanol increases the whole-cell current gradually, with maximal activation observed at 4–5 minutes after exposure (142±12% (n = 3)) of control current. After treatment with MβCD the average control current was 2 nA (Fig. 3B), and application of 50 mM ethanol increases the current to about 4 nA in 4–5 minutes (193±16% (n = 4) of pre-ethanol). This potentiation by ethanol is greater than that seen in the HEK cells before cholesterol depletion (p-value<0.01). Thus, the depletion of membrane cholesterol increases the ethanol sensitivity of BK in HEK 293 cells.

### Enantiomeric cholesterol has little effect on BK ethanol sensitivity examined in DOPE/SPM and POPE/POPS bilayers

Cholesterol's effects on BK in the severely reduced bilayer preparation can occur through: 1) modulating the biophysical properties of the lipid bilayer or 2) interacting with the channel protein or its closely associated proteins directly. Resolution of this dichotomy is critical to our understanding of the molecular basis of cholesterol modulation of ethanol action. In order to differentiate between these two possible mechanisms, we used enantiomeric cholesterol (*ent-*CHS), which has an identical chemical composition and bonding pattern (same relative configurations) as natural cholesterol. The compounds are distinguished only by their different absolute configurations. The opposite absolute configurations allow enantiomers to be distinguished by plane-polarized light or by interaction with other chiral molecules such as membrane proteins [26]. The enantiomer of natural cholesterol does not exist naturally, and must be synthesized chemically [16], [29]. Studies in the past have shown that the enantiomer has an effect similar to *nat-*cholesterol on membrane properties but differs in the ability to interact with membrane proteins [26].

**Figure 4:**
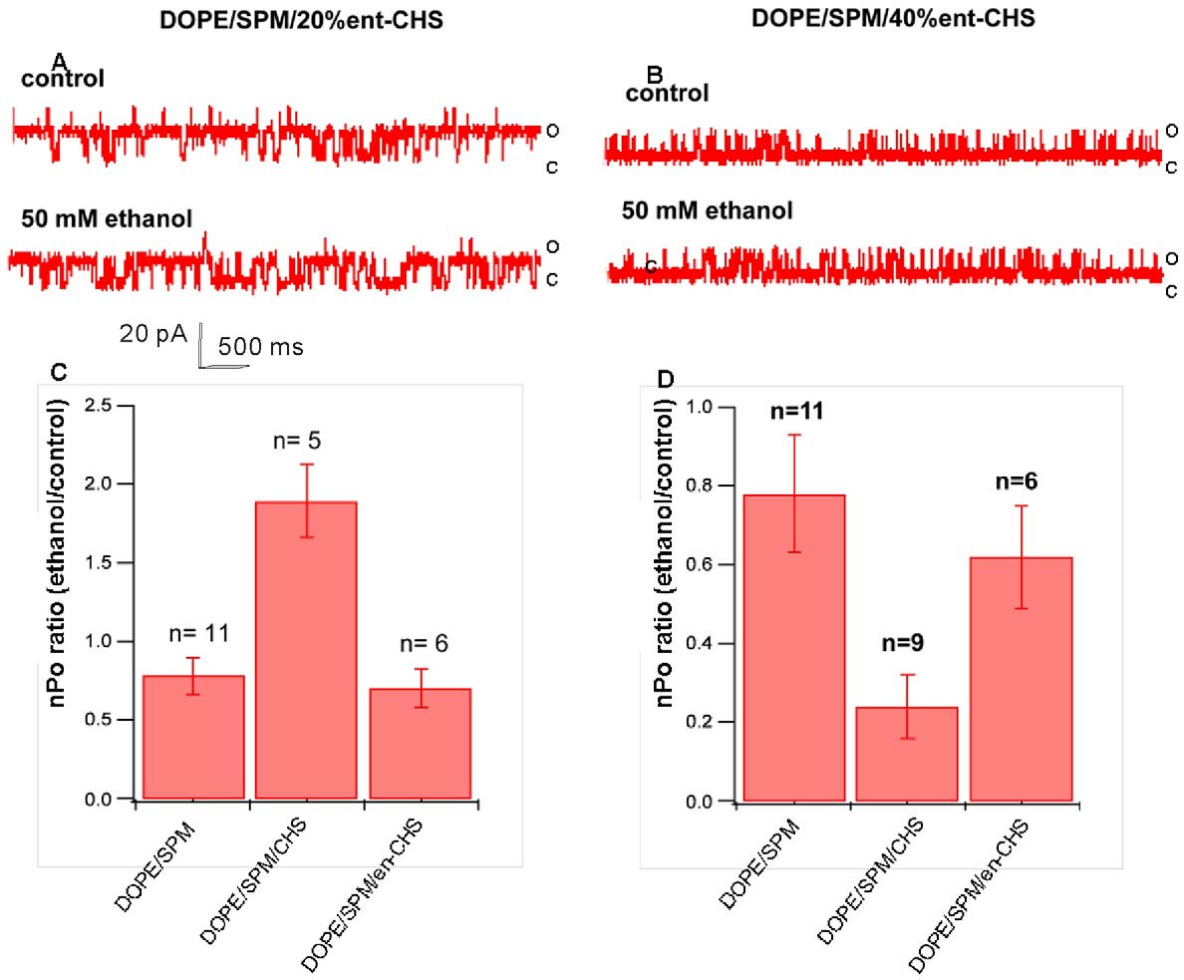
Sample traces of BK currents recorded in DOPE/SPM bilayers with (A) 20% or (B) 40% ent-CHS before and after exposure to 50 mM ethanol. (C) Compiled data showing the effect of 20% nat-CHS and and 20% ent-CHS on ethanol action. This concentration of nat-CHS introduces significant potentiation of BK activity by ethanol, whereas ent-CHS is without effect. (D) Compiled data showing the effect of 40% nat-CHS and and 40% ent-CHS on ethanol action. This higher nat-CHS concentration results in significant inhibition of channel gating by EtOH. Once again, ent-CHS is without effect.

We first tested the same concentrations of *ent-*CHS (20% and 40%) as had been used with *nat-*CHS in DOPE/SPM membranes. The average Po for BK in DOPE/SPM bilayer containing 20% *ent*-CHS and 40% *ent*-CHS are 0.55±0.12 and 0.38±0.09, which are comparable with BK channel in DOPE/SPM bilayers containing *nat*-CHS. The *ent-*CHS's effect on ethanol sensitivity of BK was examined under identical recording conditions to those for *nat-*CHS. Fig. 4 shows sample single channel recordings before and after application of 50 mM ethanol. *ent-*CHS has little effect on the ethanol sensitivity of BK, regardless of the concentration used. In each of 6 experiments with either 20% or 40% *ent-*cholesterol, exposure to 50 mM ethanol only slightly inhibited channel activity compared with ethanol's actions on BK in DOPE/SPM bilayers without *ent-*CHS. Thus, the effect of *ent-*CHS on the ethanol sensitivity of BK was dramatically different from the results obtained with *nat-*CHS.

**Figure 5:**
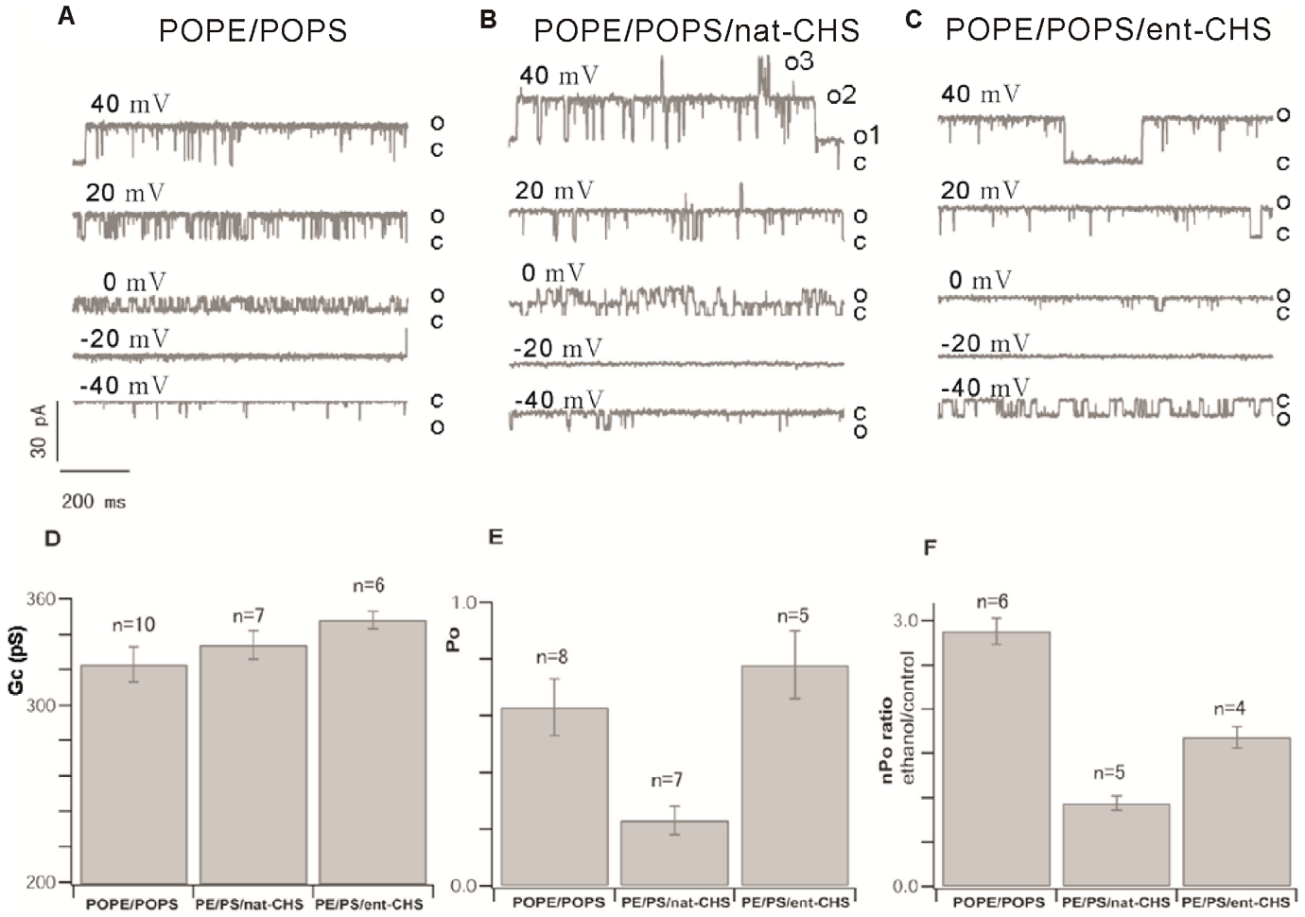
Sample single channel records of BK currents recorded in (A) POPE/POPS bilayers with (B) 20% nat-CHS or (C) 20% ent-CHS at different membrane voltages. Also shown are graphic representations of the effect of nat-CHS and ent-CHS on the (D) single channel conductance, (E) channel open probability, and (F) ethanol sensitivity. Natural cholesterol effectively reduces channel activity and ethanol sensitivity, while ent-CHS has little effect on BK channel activity but did reduce ethanol sensitivity slightly, though not as dramatically as nat-CHS did.

Next, we tested the influence of *ent-*CHS on BK channel activity in POPE/POPS bilayers, taking advantage of a large body of data examining natural cholesterol and BK in this bilayer [6]. Fig. 5 shows sample single channel traces of BK recorded in (A) POPE/POPS, (B) POPE/POPS with 20% *nat-*CHS, and (C) POPE/POPS with 20% *ent-*CHS. Addition of 20% *nat-*CHS effectively reduced channel open activity almost 3-fold, from an average of about 0.62 for BK channels in POPE/POPS bilayers to 0.23 for BK channels in POPE/POPS with *nat-*CHS. Addition of 20% *ent-*CHS to the POPE/POPS bilayer, however, did not alter channel activity (see Fig. 5E), consistent with data reported by Bukiya et al. [31]. The channel conductance, calculated from the slope of I-V plots, indicated a slight, but not statistically significant, increase in conductance by both enantiomers of CHS in POPE/POPS bilayers (Fig. 5D). We also tested the ethanol sensitivity of BKs in POPE/POPS bilayers containing 20% *ent-*CHS. The data show that 20% *ent-*CHS in POPE/POPS bilayers slightly reduced the ethanol sensitivity of BK, but significantly less than that observed for *nat-*CHS (see Fig. 5F).

### Biophysical characterization of DOPE/SPM (3/2, molar ratio) membranes with nat-CHS and ent-CHS

**Figure 6:**
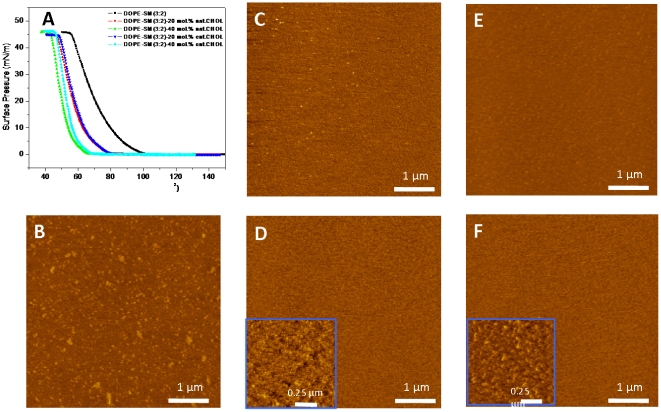
Biophysical characterization of DOPE/SPM monolayers containing cholesterol and enantiomeric cholesterol, showing that both cholesterol enantiomers have a similar influence on the biophysical properties of DOPE/SPM monolayers. (A) Isotherms of lipid monolayers of DOPE/SPM and DOPE/SPM with 20% or 40% nat-CHS and ent-CHS. Monolayers were transferred onto mica at a surface pressure of 30 mN/m and imaged with atomic force microscopy. AFM images are shown for lipid monolayers of: (B) DOPE/SPM, (C) DOPE/SPM with 20% ent-CHS), (D) DOPE/SPM with 40% ent-CHS (E), DOPE/SPM with 20% nat-CHS, and (F) DOPE/SPM with 40% nat-CHS. The bar in each image is 2 µm.

Since the number of biophysical studies showing the equivalent effects of cholesterol enantiomers on membrane properties is small, we felt that the interpretation of our data required a direct test to demonstrate that *ent-*CHS affects DOPE/SPM (3/2, molar ratio) bilayers similarly to *nat-*CHS. We carried out two sets of experiments (first with monolayers, and second, with bilayers) to confirm the validity of this assumption. In the first experiments, we spread lipid mixtures of DOPE/SPM with 20% and 40% *nat-*CHS or *ent-*CHS on the air/water interface in a Langmuir trough, to form a monolayer. We then examined the surface pressure (π) vs. area (A) isotherms for each lipid mixture (Fig. 6A). The isotherms indicate how *nat-*CHS and *ent-*CHS interact with other lipids and how they affect the phase behavior of DOPE/SPM monolayer at the air water surface [28] [46], [47]. The data (Fig. 6) show that 20% and 40% *ent-*CHS, and *nat-*CHS both effectively reduced the area per molecule in the monolayer. There is no significant difference observed in the isotherms for 20% *ent-*CHS and *nat-*CHS, but for 40% *ent*-CHS and *nat*-CHS, the isotherms showed the condensation effect of *nat*-CHS was a bit larger than that of ent-CHS. We next transferred monolayers of lipid mixtures of DOPE/SPM with 20% and 40% *nat-*CHS and *ent-*CHS onto mica and imaged with atomic force microscopy (AFM) to evaluate whether *ent-*CHS and *nat-*CHS affect the morphology of the monolayer differently (Fig. 6B–F). The DOPE/SPM (3/2) monolayer showed phase separation with many small raised domains that we assign to a SPM-enriched phase, by analogy to an earlier study [48]. Previously we had observed a mixture of small and large SPM-enriched domains for monolayers prepared in air [48]; the lack of large domains for monolayers deposited under nitrogen is consistent with reports for other lipid mixtures, in which large domains were not observed in a nitrogen environment [46].

**Figure 7:**
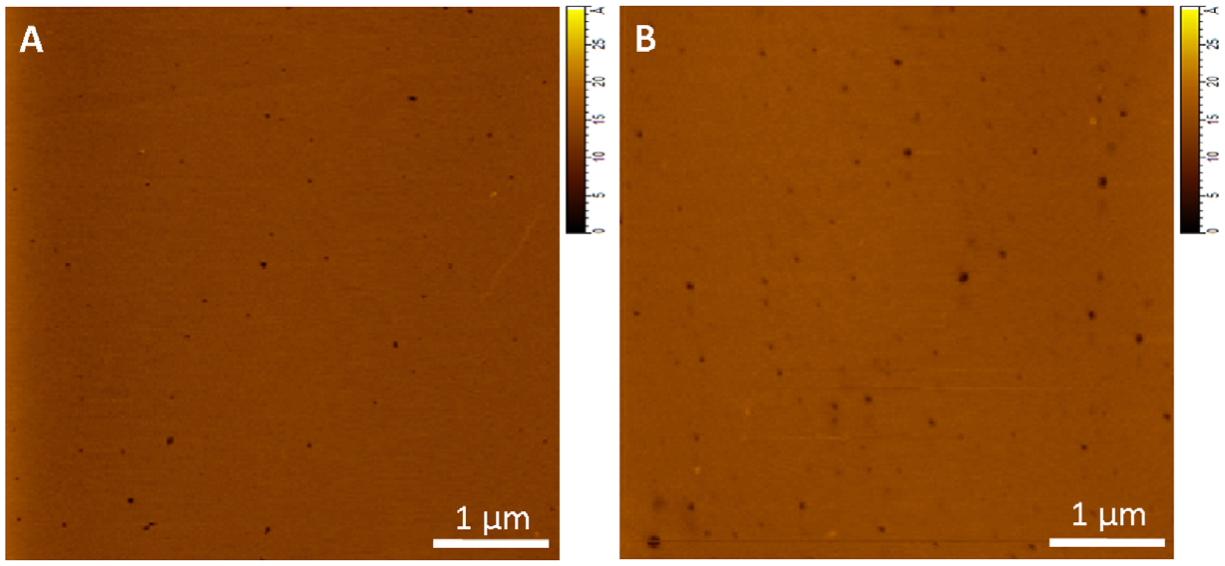
AFM images of lipid bilayers of DOPE/SPM with 20% nat-CHS (A) and 20% ent-CHS (B) prepared with vesicle deposition. Both bilayers showed some pinhole bilayer defects and there is no clear difference in topography between the two bilayers, indicating that both cholesterol enantiomers have similar effects on the DOPE/SPM bilayer.

Addition of 20 mol% *ent-*CHS or *nat-*CHS (Fig. 6 C,D) resulted in the disappearance of the small raised domains that were observed in the DOPE/SPM monolayers; similarly there was little evidence for phase separation in monolayers containing 40 mol% *nat-*CHS or *ent-*CHS. However, in this case small scale images (see inserts in Fig. 6 E, F) show that the monolayer is heterogeneous, possibly indicating the presence of small domains that are barely detectable by AFM. Interestingly, for none of these monolayers is there a significant difference in monolayer morphology between samples containing *ent-*ChS and *nat-*CHS. For the pinhole defects seen between lipid membranes containing ent-CHS (Fig. 6D) and nat-CHS (Fig. 6F), we have compared and averaged multiple 5×5 µm×µm images for several samples, obtained 29 defects/image for nat-Chol and 25 defects/image for ent-Chol, which were not statistically different. In a second set of experiments DOPE/SPM vesicles containing 20% *ent-*CHS or *nat-*CHS were used to prepare supported bilayers by vesicle fusion on mica. Bilayers were imaged by AFM to evaluate whether substitution of *nat-*CHS with *ent-*CHS had any effect on the organization of DOPE/SPM bilayers. AFM images (Fig. 7) showed uniform bilayers with a few small pinhole defects in both cases, again providing no evidence for differences between *nat-*CHS and its enantiomer.

Taken together, these data support the interpretation that *nat-*CHS and *ent-*CHS affect the bilayer similarly, and that differences observed in the actions of the enantiomeric form result primarily from an inability of *ent-*CHS to sterically interact with the protein.

## Discussion

We examined the role of cholesterol in the channel activity and response to ethanol of BK channels in POPE/POPS and DOPE/SPM lipid bilayers, as well as in the natural membrane of HEK 293 cells. We found that membrane cholesterol modulates basal function, as well as the ethanol sensitivity of the BK channel. Most interestingly, cholesterol tuning of the BK channel is a function of cholesterol concentration and of the accompanying lipids in the bilayer. Thus, lipid modulation of the physiology and pharmacology of imbedded membrane proteins is complex, with the potential for combinatorial effects. Additionally, an understanding of the underlying explanations for the actions of cholesterol on channel function and pharmacology is complicated by the fact that cholesterol's actions are mediated by both direct interactions with the protein (influenced by lipid composition of the bilayer), and by less specific actions resulting from effects within the bilayer, as discussed below. *Our results indicate that to understand membrane protein function and modulation, the protein and surrounding lipid need to be considered as a functional unit.*

### Membrane cholesterol regulates BK channel activity

Cholesterol plays an essential role in the formation and maintenance of signaling micro-domains known as lipid or membrane rafts. BK channels often reside in sphingomyelin- and cholesterol-rich raft-like caveolae domains [20], [49], such as in bovine aortic endothelial cells [50], rat uterine myocytes [51] and human melanoma IGR39 cells [45]. Changes in cholesterol content in cell membranes have been shown to effectively alter BK channel activity and lead to a dramatic change of BK activity. Depletion of membrane cholesterol in glioma cells [25] and in rat uterine myocytes [51] significantly reduces BK current. On the other hand, the activity of BK channels in bovine aortic endothelial cells [50] and in pituitary GH3 cells [20] is enhanced by the depletion of membrane cholesterol. The data we present here show that in HEK 293 cells, the depletion of membrane cholesterol by treatment with MβCD increases BK currents 2–4 fold. Membrane cholesterol in HEK 293 cells is in the range of 30–50% (molar ratio) of total lipids (*Lasalde-Dominicci*, personal communication). Treatment with MβCD removes membrane cholesterol and disrupts lipid rafts. Membrane cholesterol is reduced about 51±28% by treatment with 10 mM MβCD for 60 minutes [52], and 41% by treatment with 5 mM MβCD for 30 minutes [45]. In our experiments, we found that depletion of membrane cholesterol with 5 mM MβCD produced maximal increases in BK channel activity within 10–15 minutes (see Fig. 2D). Furthermore, when BK channels were isolated from transfected HEK 293 cells and reconstituted into lipid domain-containing DOPE/SPM bilayers, systematically increasing cholesterol content (from 10% to 40% of total lipids, molar ratio) reduced channel activity (see Fig. 1D), consistent with the increased activity observed in the natural HEK 293 membrane when cholesterol was reduced. Thus, although BK channels in HEK 293 membrane are certain to reside in different lipid environments than BK channels in the artificial lipid bilayer, the cholesterol effect on BK channel activity observed in HEK 293 cells is retained in the artificial membranes. *This suggests that cholesterol's interaction with the channel protein is preserved even when moving from reduced to complex membrane environments.*

It has been well documented in the literature that BK channels can reside in lipid rafts. We believe that our data allow a connection to be drawn between results from cells where BK channels are located in “cholesterol/SPM-enriched rafts”, and the different outcomes from POPE/POPS vs. DOPE/SPM bilayers. The validity of this interpretation depends upon a demonstration of the location of BK channels in raft vs. raft-free HEK cells in the absence and presence of the dextrin/ethanol. Indeed, these experiments have been ongoing, and indeed, ethanol is capable of translocating the channel protein between raft and non-raft membrane (manuscript in preparation). Our interpretation of data is dependent upon the dispersion of lipids accompanying the channel protein when placed into the planar bilayer. Several pieces of evidence previously published [53] indicate that exchange between native lipid associated with the incorporated channel exchanges with the planar bilayer lipid: “First, BK channels were modulated by the amount of fixed charge present in the bilayer, with channels in neutral PE bilayers exhibiting lower *Po* and conductance values than channels in negatively charged PE/PS bilayers [53]. These data are qualitatively identical to those of Moczydlowski *et al.* (1985) and indicate that replacement of native with bilayer lipid is extensive, possibly complete. This interpretation is buttressed by additional evidence in the literature. For example, the activity of nystatin, a peptide that requires ergosterol to form channels, is lost when membrane vesicles containing nystatin and ergosterol are incorporated into ergosterol-free membranes, presumably because of diffusion of ergosterol away from the channel complex (Woodbury and Miller, 1990). ESR studies of reconstituted nicotinic acetylcholine receptors indicate that the lipid at the protein/lipid boundary is relatively motionally restricted, but, nevertheless, can exchange with the bulk lipid. This exchange rate is rapid, on the order of 107/sec, and is slowed by high protein/lipid ratios (Ellena *et al.*, 1983; Barrantes, 1989). We might expect this exchange to be faster in our system because the protein/lipid ratio is likely to be far lower than in biological membranes. Thus, the data strongly suggest that the bilayer lipid substitutes for the native lipid immediately surrounding incorporated channels, greatly reducing the level of transverse and lateral membrane heterogeneity.

### Specific cholesterol-BK interactions contribute to cholesterol's influence on BK channel activity and ethanol action

We used enantiomeric cholesterol (*ent-*CHS, the mirror image compound of natural cholesterol (*nat-*CHS)) to probe the structural specificity of cholesterol's influence on BK physiology and ethanol pharmacology. Atomic force microscopy (AFM) and Langmuir monolayer techniques were used to test the assumption that enantiomeric forms of cholesterol are equivalent in their effects on the biophysical properties of these lipid membranes. The data show that while *ent-*CHS is not significantly different from *nat-*CHS in affecting the biophysical properties of the bilayers, it has a dramatically different effect on BK channel activity and ethanol sensitivity in comparison with *nat-*CHS.

In the absence of cholesterol (*nat-*CHS or *ent-*CHS), the BK channel exhibits potent potentiation during exposure to 50 mM ethanol in a POPE/POPS bilayer, whereas the channel is inhibited by the same concentration of ethanol in a DOPE/SPM bilayer [8]. Insertion of increasing amounts of *nat-*CHS into a POPE/POPS bilayer is associated with linearly increasing suppression of ethanol activation of the BK channel [6]. Data in Fig. 1B indicate that in a DOPE/SPM bilayer, the insertion of low concentrations (20%, molar ratio) of cholesterol supports ethanol activation of BK activity, absent in the DOPE/SPM bilayer lacking cholesterol. Insertion of higher concentrations (40% molar ratio) of cholesterol into the DOPE/SPM bilayer, however, leads to strong ethanol inhibition of the BK channel. Thus, the modulation of ethanol action by insertion of cholesterol into lipid bilayers is complex, showing dependency on cholesterol concentration as well as on the accompanying lipids (discussed later). When we replaced *nat-*CHS with an equal amount of *ent-*CHS, we observed that in the DOPE/SPM bilayer, *ent-*CHS had no effect on ethanol sensitivity, while in the POPE/POPS bilayer, *ent-*CHS had an effect, but significantly smaller than that of *nat-*CHS. These results suggest that specific cholesterol-BK interactions underlie cholesterol's influence on BK channel activity [31], as well as ethanol action on BK. Since the simple bilayer preparation is devoid of complex membrane or intracellular organizing elements, the differences observed between the enantiomers of cholesterol in affecting BK channel function and ethanol pharmacology suggest a specific interaction of cholesterol with the BK channel protein. Since *nat*- and *ent*-Chol have a similar effect on the bilayers tested in the absence of BK and ethanol, it is unlikely that their effect on the BK channel is due to altering bilayer properties. However, full confirmation that cholesterol's influence on BK's ethanol response results from a specific interaction between cholesterol and the channel protein will require more compelling evidence that *nat*- and *ent*- chol behave similarly in the presence of the BK channel and ethanol. However, the results of this manipulation will not be easy to interpret, because of the complexity resulting from the combined influence of *ent*-CHS and *nat*-CHS on the lipid bilayer, plus the influence of a specific *ent*-CHS- or *nat*-CHS-BK protein interaction on the lipid bilayer.

Cholesterol has been shown to interact directly with many membrane proteins important for neural function [26], [31], [54]. The data we present here show further a dramatic difference between *ent-*CHS and *nat-*CHS on the ethanol sensitivity of BK in DOPE/SPM and POPE/POPS bilayers, providing direct evidence that cholesterol's effect on BK channel activity and ethanol action might be via a direct interaction with the BK protein. The first clue that cholesterol influences ethanol's actions on BK via a direct interaction with the channel protein came from a previous study showing that cholesterol could effectively antagonize ethanol's action on BK in a minimal POPE/POPS artificial lipid bilayer lacking both membrane complexity and intracellular components [6]. Modulation of BK channel function by CHS and ethanol is characterized by striking similarities in POPE/POPS bilayers: both agents modify channel Po with minor, if any, modification of conductance. Furthermore, kinetic analysis showed that cholesterol's effect on single channel activity parameters (such as open and closed times) mirror that of ethanol, indicating that cholesterol and ethanol may target common hslo channel dwelling states.

### Cholesterol's modulation of ethanol action is dependent upon accompanying lipids

As we discussed earlier, the modulation of ethanol action by insertion of cholesterol into lipid bilayers is complex, showing dependency not only on cholesterol concentration but also on the accompanying lipids. How can we account for the smaller, but still observable effect of *ent-*CHS in the POPE/POPS bilayer but not in DOPE/SPM bilayer? Though our results suggest that cholesterol influences BK channel function and ethanol pharmacology through a direct cholesterol-BK interaction, the insertion of cholesterol into lipid bilayers will alter the biophysical properties of the bilayer, which will also affect BK function. Firstly, insertion of cholesterol can condense lipid membranes, increasing lateral stress [55]. The increase in lateral stress can favor the open or closed state of the channel residing in the bilayer, and consequently change the channel activity of the channel. Also, insertion of cholesterol into the lipid bilayer alters the thickness of the bilayer [56], which regulates BK channel activity and modulates ethanol sensitivity of BK [32]. Therefore, cholesterol's modulation of the BK channel and ethanol action through modulating the biophysical properties of the lipid bilayer is likely. Indeed, the idea that ethanol's actions on membrane proteins derive primarily from perturbation of membrane lipids dominated thinking in the ethanol molecular pharmacology field for years [6], [44], [56].

Secondly, insertion of cholesterol can alter lipid phase distribution within the bilayer. Previous studies have shown that the phase status within lipid bilayers is influenced by the inclusion of cholesterol in a concentration-dependent manner [33], [47]. Thus, the concentration-dependent cholesterol modulation of BK ethanol sensitivity in DOPE/SPM bilayers might be explained if we consider that: 1) the DOPE/SPM bilayer is a phase separated lipid bilayer, containing a DOPE-rich liquid-expanded (LE) phase and a SPM-rich gel domain [48], and 2) cholesterol can form lipid rafts with sphingomyelin in the DOPE/SPM bilayer.

At low concentrations, the insertion of cholesterol into the DOPE/SPM bilayer resulted in ethanol-mediated activation of BK. Previous work showed that in phase separated DOPC/SPM membranes, cholesterol at lower concentrations partitions primarily into the DOPC-dominated phase, while at higher concentrations, cholesterol mostly partitioned into the SPM-dominated domain [33]. By analogy, in the current work, we might expect that lower amounts of cholesterol introduced into the phase-separated DOPE/SPM (3∶2, molar ratio) bilayer will mostly partition into the DOPE phase. The inserted cholesterol will condense the DOPE phase, increasing its thickness, and diminishing phase separations within the membrane (as seen in AFM images, Figs. 6). Thus, in the presence of low concentrations of cholesterol, the reduced domain structure becomes similar to the state seen in bilayers of previously studied DOPE/PCs (PCs from chain length of 18 to 24 carbons), where ethanol was similarly seen to activate BK [8]. Insertion of higher concentrations of cholesterol (above 30%, molar ratio) into DOPE/SPM bilayers, however, leads to the formation of SPM-cholesterol domains in a liquid ordered (L_O_) phase [33], a structure that has been designated as a lipid raft [57]. Previous work has shown that in lipid bilayers containing ordered lipid domains, ethanol inhibits BK activity [8].

In contrast to the DOPE/SPM bilayer, the POPE/POPS bilayer is in a homogeneous LE phase, and because SPM is lacking, cholesterol cannot form an ordered lipid raft in the POPE/POPS bilayer. Insertion of increasing concentrations of cholesterol into a POPE/POPS bilayer will progressively increase the lateral pressure within the bilayer, which will reduce the ethanol sensitivity of BK in the bilayer [6]. The partial effectiveness we observed for *ent-*CHS in the POPE/POPS bilayer, absent in the DOPE/SPM bilayer, is likely due to greater fluidity in the POPE/POPS bilayer. It is conceivable that the ordered lipid phase (rigid bilayer) of the DOPE/SPM bilayer imposes greater structural specificity than the less ordered (or disordered) phase (fluid bilayer) of the POPE/POPS bilayer, where the steric constraint of the cholesterol-BK interaction would be lessened due to the greater flexibility in movement or rotation of cholesterol. This scenario would explain why replacement of *nat-*CHS with *ent-*CHS in DOPE/SPM bilayers resulted in total loss of influence on ethanol's action on BK, while replacement of *nat-*CHS with *ent-*CHS in the POPE/POPS bilayer leads to a less complete effect on ethanol action.

In summary, we show that cholesterol regulates BK channel activity and its ethanol sensitivity in a complex manner, which likely involves both a direct interaction with the channel protein and alteration of biophysical properties of accompanying bilayer lipids. It has been suggested that cholesterol plays a crucial role in the development and maintenance of neuronal function and plasticity [58], [59]. Failure of cholesterol homeostasis results in synaptic degeneration [60], [61], and variations in cholesterol levels are closely associated with neurological disorders such as Alzheimer's disease [62]. BK in neuronal membranes resides primarily in cholesterol-rich rafts [45]. Thus, our bilayer data suggesting the importance of direct cholesterol-BK interaction in regulation of ethanol action may have significant implications for our understanding of molecular modulation in complex biological membranes. Membrane cholesterol concentrations are altered during the formation and dissociation of lipid rafts. Therefore, the concentration-dependency of cholesterol's modulation of channel function and inhibition of ethanol action on BK can be regulated through the formation and disruption of lipid rafts, and by movement of the channel into or out of rafts. Tolerance to ethanol at the molecular level has been termed molecular tolerance. Acute tolerance of BK is observable within minutes of exposure to the drug [5]. The possibility that ethanol tolerance is mediated by cholesterol and altered cholesterol concentrations within individual lipid rafts is an appealing potential mechanism for the changes in ethanol sensitivity characteristic of molecular tolerance. Finally, BK channels examined in this study contain only the alpha subunit. The BK beta subunit is known to influence ethanol's actions, including molecular tolerance [63] and is often complexed with the alpha subunit in neurons. Thus, in light of the findings presented here, it will prove very interesting to further pursue these studies with BK channels of varied alpha and beta subunit composition.
